# Towards controlling activity of a peptide asparaginyl ligase (PAL) by lumazine synthetase compartmentalization

**DOI:** 10.1039/d4fd00002a

**Published:** 2024-02-20

**Authors:** T. M. Simon Tang, Louis Y. P. Luk

**Affiliations:** a School of Chemistry & Cardiff Catalysis Institute, Cardiff University Main Building, Room 1.54, Park Place Cardiff CF10 3AT UK lukly@cardiff.ac.uk

## Abstract

Peptide asparaginyl ligases (PALs) hold significant potential in protein bioconjugation due to their excellent kinetic properties and broad substrate compatibility. However, realizing their full potential in biocatalytic applications requires precise control of their activity. Inspired by nature, we aimed to compartmentalize a representative PAL, OaAEP1-C247A, within protein containers to create artificial organelles with substrate sorting capability. Two encapsulation approaches were explored using engineered lumazine synthases (AaLS). The initial strategy involved tagging the PAL with a super-positively charged GFP(+36) for encapsulation into the super-negatively charged AaLS-13 variant, but it resulted in undesired truncation of the enzyme. The second approach involved genetic fusion of the OaAEP1-C247A with a circularly permutated AaLS variant (cpAaLS) and its co-production with AaLS-13, which successfully enabled compartmentalization of the PAL within a patch-work protein cage. Although the caged PAL retained its activity, it was significantly reduced compared to the free enzyme (∼30–40-fold), likely caused by issues related to OaAEP1-C247A stability and folding. Nevertheless, these findings demonstrated the feasibility of the AaLS encapsulation approach and encourage further optimization in the design of peptide-ligating artificial organelles in *E. coli*, aiming for a more effective and stable system for protein modifications.

## Abstract 2

We aim to compartmentalize a representative peptide asparaginyl ligase, OaAEP1-C247A, within protein containers to create artificial organelles with substrate sorting capability.

## Graphical abstract

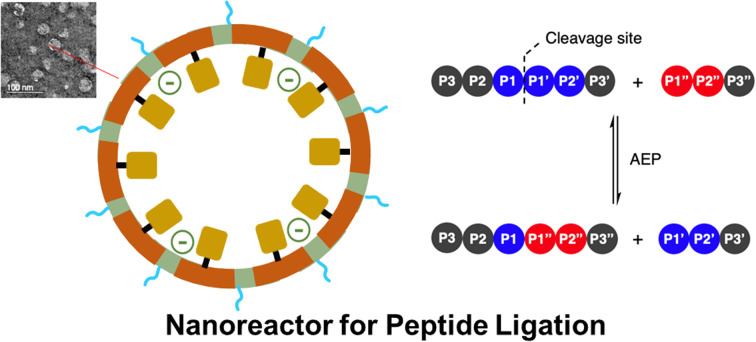

## Introduction

**Fig. 1:**
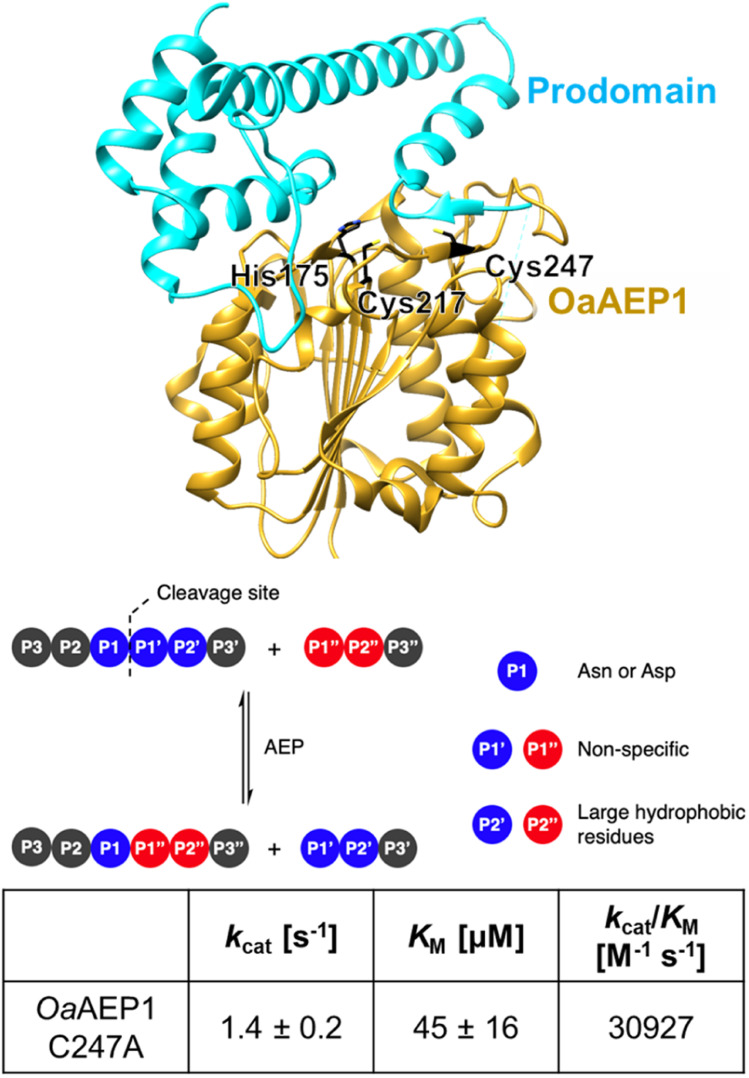
OaAEP1-C247A is a representative PAL featuring a relaxed substrate scope, at the P1′ and P1′′ sites, and excellent kinetic properties (parameters at pH 7.5 and 25 °C obtained from ref. 12). Model built from wild-type OaAEP1 (PDB = 5H0I) with the N-terminal core (yellow), C-terminal cap domain (cyan), catalytic residues His175 and Cys217 as well as the gate residue Cys247 highlighted (black).

OaAEP1-C247A represents a class of plant transpeptidases called peptidyl asparaginyl ligase (PAL) that have promising potential in the biocatalytic production of modified peptides and proteins (Fig. 1).^1–16^ In nature, these enzymes play roles in protein processing, including albumin maturation,^17^ and syntheses of cyclic peptides, such as Kalata B1.^18^ Because of their excellent kinetic properties and short recognition sequences, PALs have also been used in laboratories to cyclize various peptides and proteins; these include ones originating from plants, such as sunflower trypsin inhibitor 1 (ref. 15) and MCoTI-II,^19^ as well as non-native substrates, including GFP,^20^ somatropin,^20^ MSP2 (ref. 11) and the p53 binding domain.^21^ Further analysis revealed that OaAEP1-C247A has significantly broad substrate scope at the P1′′ position, accepting different primary and secondary amine nucleophiles for reactions, and hence they have been extensively used in protein terminal modifications.^9^ In parallel, PALs have also been used in segmental synthesis of proteins generating isotopically labelled hybrids.^22^ These experiments have collectively marked PALs as invaluable tools in biochemical and biotechnological research.

**Fig. 2:**
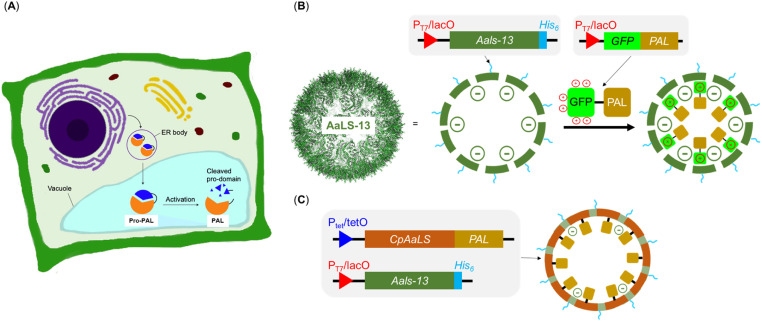
Strategies for introducing guest protein PALs into engineered lumazine synthase protein cages as artificial organelles. (A) Endoplasmic reticulum and vacuole are the organelles used to compartmentalize PALs in plant cells; (B) AsLS-13, modelled from PDB 5MQ7, is a negative supercharged variant that can encapsulate cargoes with a net positive charge; (C) the circularly permutated AaLS (cpAaLS) could encapsulate cargo through genetic fusion.

Though the relatively short and non-specific substrate recognition profile of PALs are appealing for applications, it may also complicate its production due to its potential for undesired side-reactions in the expression host.^13,14^ In nature, the activity of PALs is strictly regulated through cap-domain protection as well as spatial compartmentalization within organelles. In plants, PALs are often compartmentalized in the endoplasmic reticulum (ER) bodies with a cap domain masking the active site. In response to environment stimuli, the PALs are delivered to the vacuole, another plant organelle, where the acidic environment triggers autocatalytic removal of the cap domain for enzyme activation (Fig. 2A).^17^ Similarly, the mammalian counterparts, known as legumain and recognized as targets in cancer therapy, reside in the ER and become activated under specific conditions.^23,24^ This organization ensures that only substrates with the appropriate localization sequences can access these organelles and undergo modifications only when PALs are activated. This multi-layered regulatory mechanism suggests that PAL can be cytotoxic because it can potentially modify non-targeted asparagine residues of cytoplasmic proteins. Indeed, gene expression of PALs in laboratories often include the cap domain keeping them in their dormant form,^1,9,12–14^ but compartmentalization and substrate sorting are missing due to the absence of organelles in *E. coli.* Nevertheless, compartmentalization may allow catalytically active PAL to be produced in *E. coli* without the need of enzyme activation, simplifying its production process. Furthermore, substrate sorting through compartmentalization will allow precise activity control of PAL, a feature that will likely find uses in the process of screening engineered PALs and/or active ligated peptide products.

The engineered lumazine synthase from *Aquifex aeolicus* (AaLS) can self-assemble to generate protein cages that can encapsulate specific enzymes, effectively transforming them into artificial organelles in *E. coli* for activity control.^25–35^ One notable variant, AaLS-13, features a supercharged negative cavity and a porous architecture, which enable uptake of enzymes and proteins carrying positively charged tags, including Tobacco Etch Virus protease (TEVp), retroaldolase, β-lactamase, cyclohexylamine oxidase, catalase-peroxidase, NADH oxidase, aldehyde dehydrogenase and monoamine oxidase and ferritin (Fig. 2B).^28,31^ In particular, the substrate sorting capability was demonstrated using a compartmentalized TEVp system, in which the anionic environment of AaLS-13 enables selective cleavage of peptide substrates with a net positive charge.^28^ Further innovation was achieved through circular permutation of the wild-type AaLS gene (cpAaLS), which repositioned the termini from cage exterior to interior, thereby enabling the compartmentalization of protein cargo by genetic fusion (Fig. 2C).^27^ Importantly, cpAaLS can co-assemble with the subunit of AaLS-13, resulting in a patchwork cage that can potentially facilitate cargo internalization *via* both genetic fusion and electrostatic interactions.

Here, we have recruited lumazine synthase technologies to create compartmentalized PALs to create a nanoreactor with substrate sorting capabilities, which have the potential to be transformed into artificial organelles. We observed that fusing both the OaAEP1-C247A to the terminus (N or C) of the positively charged tag GFP(+36) resulted in truncation products. When the gene of a cap domain-free OaAEP1-C247A fused to the C-terminus of cpAaLS and co-expressed with AaLS-13, a patchwork cage encapsulating PAL was successfully made, as verified by size exclusion chromatography (SEC), SDS-PAGE and transmission electron microscopy (TEM) analyses. Activity of the caged PAL was demonstrated by a FRET-based kinetic assay and was lower than that of the free enzyme. For future work, we aim to improve activity of the caged PAL, aiming to make an artificial organelle with substrate sorting capacity.

## Results and discussion

**Fig. 3:**
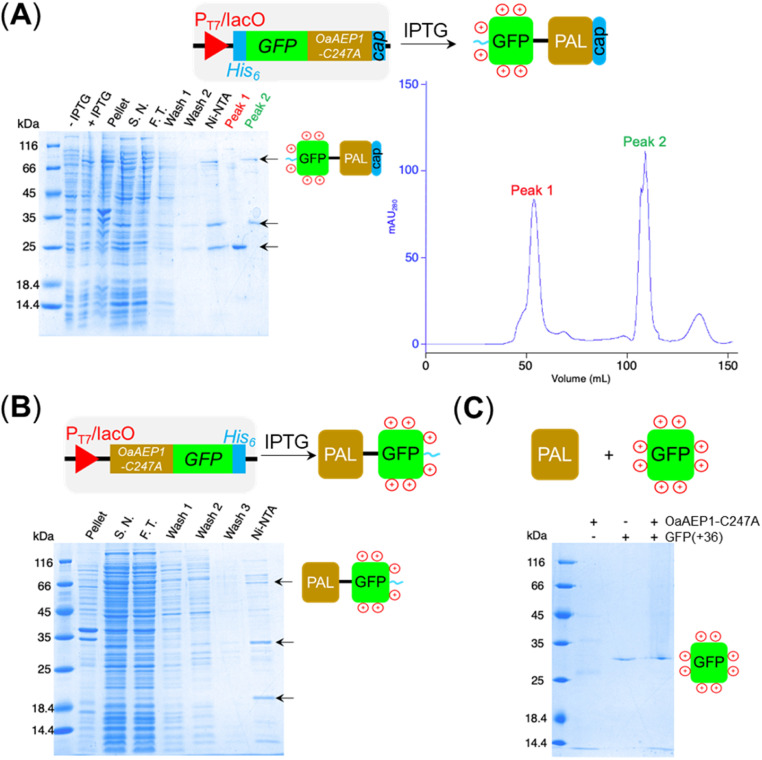
(A) SDS-PAGE analysis of recombinant GFP(+36)-OaAEP1-C247A. Arrows indicate bands corresponding to the protein of interest, GFP(+36)-OaAEP1-C247A and proposed truncation fragments. SDS-PAGE lanes: *E. coli* culture before IPTG addition (-IPTG), after IPTG addition (+IPTG), insoluble fraction of lysate (pellet), soluble fraction of lysate (S.N.), flow through (F.T.) from Ni-NTA, wash fractions (wash 1 and 2), protein elution (Ni-NTA), protein elution from SEC (peak 1 and 2), colour coded lanes correspond to the peaks in the chromatogram. (B) SDS Analysis of recombinant OaAEP1-GFP(+36) in which the protein constructs were swapped. (C) GFP(+36) stability towards OaAEP1-C247A treatment. The reaction was conducted in 50 mM MES buffer (pH 6.0) added with 50 mM NaCl, 1 mM EDTA and 0.5 mM TCEP. The final concentration of GFP(+36) was fixed at 4 μM with OaAEP1-C247A at either 2 or 0 μM. The mixture was incubated at 20 °C for 22 h prior to SDS-PAGE analysis.

Aiming to create a PAL construct which can be encapsulated by AaLS-13 capsids, the genes of OaAEP1-C247A core and cap domain, combined using a flexible GS linker (GSGGSGGSGGSGGSS), were appended downstream and in frame to that of the positively charged tag, His_6_-GFP(+36) (Fig. 3A, see also Experimental for construct sequence). This newly assembled gene enabled the production of His_6_-GFP(+36)-OaAEP1 by *E. coli*. Recombinant gene expression was induced by IPTG in *E. coli* BL21 (DE3) and the protein of interest was purified by Ni^2+^-nitrilotriacetic acid immobilized metal ion affinity chromatography (Ni-NTA IMAC), followed by size exclusion chromatography (SEC). The protein containing fractions after each step were analyzed by SDS-PAGE. Upon IPTG-induced gene expression, a band corresponding to the full length GFP(+36)-OaAEP1 fusion protein (79.6 kDa) was observed. Although the cap domain was expected to be retained because the fusion construct had not been treated with acidic buffer for enzyme activation, a significant degree of undesired protein truncation was detected with distinctive bands at around 25 and 30 kDa were detected, which likely corresponded to AEP and GFP, respectively. Two proteins were isolated in the subsequent purification by SEC. Unexpectedly, the first eluted peak (1) corresponded to the smaller protein (25 kDa), while the later peak (2) corresponded to the larger protein (35 and 66 kDa; Fig. 3A). The shorter retention time observed for the lower molecular weight protein suggests the presence of quaternary PAL structures, which are maintained through non-covalent interactions. Similarly, mass spectrometry analyses revealed fragments close to the truncated bands (25 663 and 39 875 Da), but the original fusion construct was not detected.

To test whether protein truncation can be avoided, the position of the supercharged protein and OaAEP1-C247A was swapped. In this alternative construct, the core domain of OaAEP1-C247A was positioned upstream to a C-terminal His_6_-tagged GFP(+36). A shorter tripeptide spacer 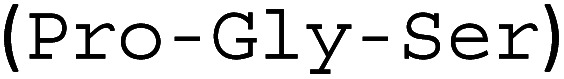 was used to try to avoid unwanted truncation (see Experimental for construct sequence). Upon IPTG-induced gene expression in *E. coli* BL21(DE3) and Ni-NTA IMAC purification, the OaAEP1-GFP(+36) fusion construct was obtained (63 kDa) but also appeared to be a faint band in SDS-PAGE analysis (Fig. 3B). Instead, the elution predominantly generated bands of truncated proteins. Short, positively charged peptide tags such as Arg_10_ were initially used to load cargo proteins into the AaLS-13 capsids.^36^ However, such tags were predicted to be ineffective in creating a net positive charge overall, as they are particularly prone to proteolysis. Furthermore, the core domain of OaAEP1-C247A contains a large number of negatively charged residues with an estimated pI of 5.11, and hence a short positive tag will unlikely be sufficient for substrate sorting.

PALs, often referred to as “asparaginyl endopeptidase (AEP),” were originally characterized as a protease because they can hydrolyze the C-terminal peptide bond of internal asparagine and aspartate residues, especially when an ideal nucleophile is absent.^1,9,12–14,16,18^ Indeed, self-cleavage of PALs is well-documented. Furthermore, the primary structure of GFP(+36) contains multiple recognition sequences compatible for OaAEP1-C247A recognition (see Experimental for its sequence).^37^ Accordingly, we prepared the fluorescent protein independently, treated with free OaAEP1-C247A and conducted tests to assess for potential proteolysis. No degradation was detected by SDS-PAGE analysis after incubation with OaAEP1-C247A overnight (21 h) at 20 °C (Fig. 3C). Accordingly, truncation could be caused by enzyme fusion, which could drive hydrolysis as a consequence of proximity effect. Alternatively, the truncation of the fusion construct could be caused by an unidentified protease produced by the recombinant host *E. coli* BL21(DE3).

**Fig. 4:**
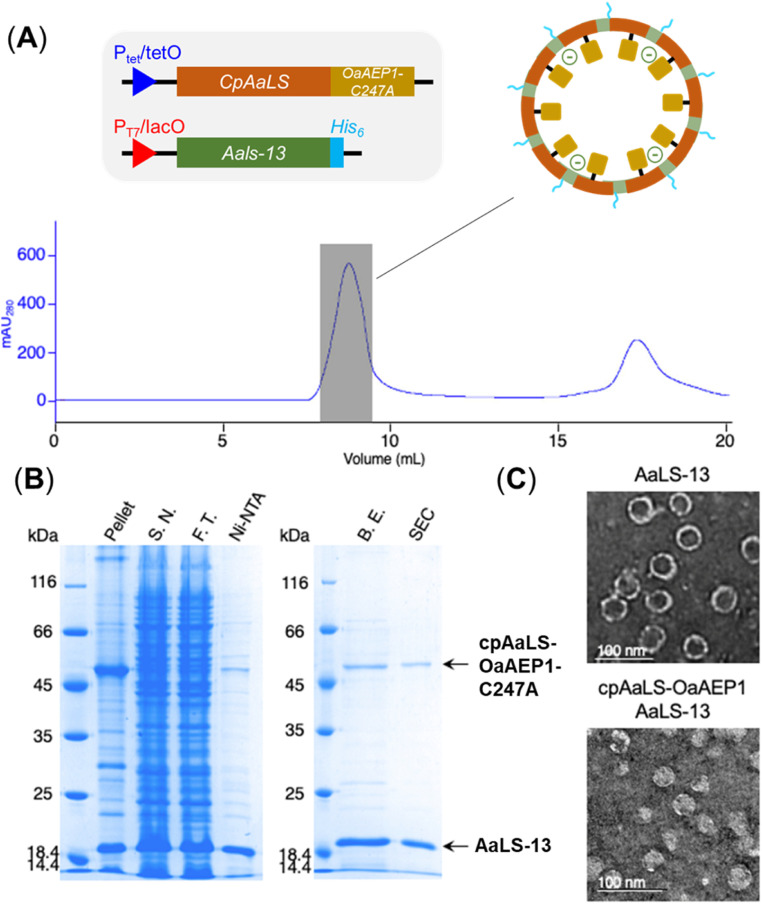
Production of cpAaLS/AaLS-13 protein cage encapsulated with OaAEP1-C247A. (A) Size exclusion chromatogram for the protein mixture. Grey shaded area corresponds to the patchwork cage. (B) SDS-PAGE analysis of fraction obtained during purification. Arrows indicate bands corresponding to the protein of interest, cpAaLS-OaAEP1 and AaLS-13. SDS-PAGE lanes: insoluble fraction of lysate (pellet), soluble fraction of lysate (S.N.), flow through (F.T.) from Ni-NTA IMAC, protein elution from Ni-NTA IMAC (Ni-NTA), imidazole removed by buffer exchange (B.E.), protein elution from SEC (SEC). (C) TEM images of empty AaLS-13 and loaded patchwork capsids. Scale bar = 100 nm.

Since the production of a positively tagged fusion construct was unsuccessful, another encapsulation approach based on the use of circularly permutated AaLS variant (cpAaLS) was explored. The N and C-termini of cpAaLS were moved from the exterior surface to the interior of the protein cage,^27^ and hence it can be genetically fused with an enzyme such as OaAEP1-C247A and co-produced with AaLS-13, affording a patchwork protein cage with the PAL incorporated in the cavity. Following an established experimental protocol,^27^ the gene encoding the core domain of OaAEP1-C247A was appended downstream of the gene of cpAaLS with a AGGAGGS linker (see Experimental for sequence). Expression of this gene construct was controlled by a tetracycline ON expression system, which offered orthogonal control to the IPTG-induced gene expression for AaLS-13. When produced in *E. coli* BL21(DE3), the fusion construct was found to aggregate in inclusion bodies. We attributed this observation to the mis-folding of OaAEP1-C247A, which possesses a cystine pair within the enzyme core. Hence, the gene of the cpAaLS fusion construct was transformed into SHuffle® T7 Express and co-expressed with the gene of the His_6_-tagged AaLS-13. Although a significant portion of cpAaLS-OaAEP1 appeared in the pellet, the fusion protein was successfully isolated from Ni-NTA IMAC as indicated by SDS-PAGE analysis, suggesting its association with the His_6_-tagged AaLS-13. When the protein mixture was further purified by SEC, the fusion construct was eluted with the characteristically short retention time suggesting formation of the patchwork cage (Fig. 4A). The SDS-PAGE analysis of the SEC-purified protein revealed the presence of two proteins corresponding to the size of AaLS-13 and cpAaLS-OaAEP1-C247A, with no truncation detected (Fig. 4B). The formation of loaded protein capsids was confirmed by negative stain TEM, where filled circular particles with diameters around 40 nm were observed (Fig. 4C). Nevertheless, LC-MS analysis of the patchwork cage did not reveal a construct close to the expected size (48 kDa).

**Fig. 5:**
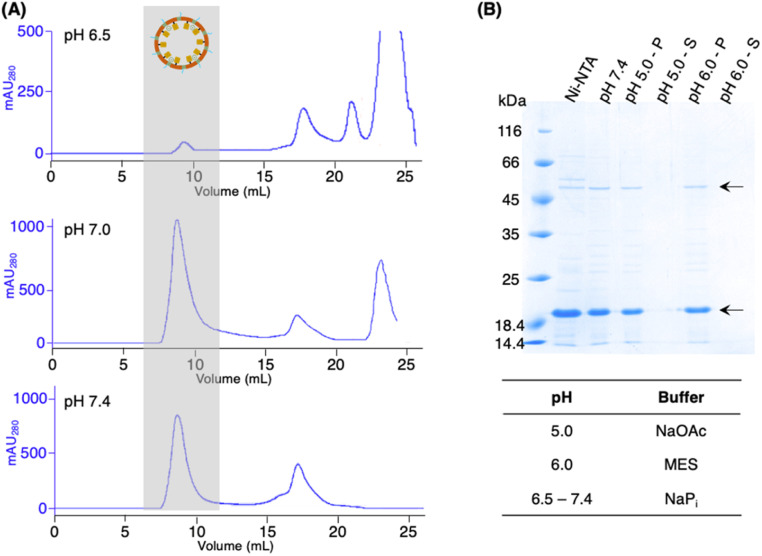
(A) SEC of the patchwork cage cpAaLS-OaAEP1-C247A/AaLS-13 was performed in buffer at pH 6.5, 7.0 and 7.4. (B) AaLS capsids obtained from SEC (pH 7.4) were acidified by buffer exchange. The resulting solution and precipitates were analysed by SDS-PAGE. Arrows indicate the bands corresponding to cpAaLS-OaAEP1-C247A and AaLS-13. SDS-PAGE lanes: protein elution from Ni^2+^-NTA (Ni-NTA), protein elution from SEC (pH 7.4), precipitate after buffer exchanged to pH 5.0 (pH 5.0 – P), soluble fraction after buffer exchanged to pH 5.0 (pH 5.0 – S), precipitate after buffer changed to pH 6.0 (pH 6.0 – P), soluble fraction after buffer changed to pH 6.0 (pH 6.0 – S).

While the activity PAL is known to be optimal in acidic conditions,^1,9,12–14,16,18^ AaLS-13 capsids were suggested to be prone to precipitation at pH < 7.0.^32,33,36^ Hence, the pH stability of the patchwork cage (cpAaLS-OaAEP1-C247A/AaLS-13 complex) was investigated. The protein cages prepared above were subjected to SEC analysis in buffer at different pH (6.5, 7.0 and 7.4). While a large quantity of the cage complex (∼9 mL) was obtained in the pH 7.0 and 7.4 chromatograms, the corresponding absorbance peak reduced significantly at pH 6.5 (Fig. 5A). When the cage complex was exchanged with acidic buffers (pH 5.0 and 6.0), precipitation was observed. Analysis by SDS-PAGE indicated that the proteins of interest were present in the insoluble precipitate, rather than the supernatant (Fig. 5B). The findings reported here confirmed that AaLS-13 protein cages are unstable in acidic conditions. Consequently, enzyme activity studies were performed at pH 7.0 as OaAEP1-C247A remains active at neutral and slightly alkaline pH.

**Fig. 6:**
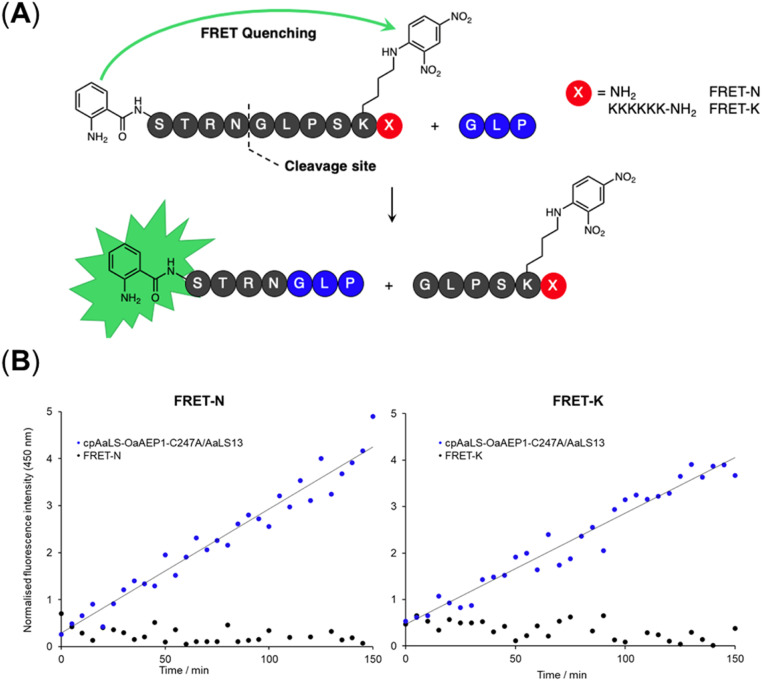
Fluorescence activity assay for the free and encapsulated OaAEP1-C247A towards FRET-N and -K. (A) Schematic of AEP-mediated ligation resulting in the separation of the FRET donor and acceptor pair. Position X can be either the C-terminal amide or a positively charged Lys_6_ tag for the neutral (FRET-N) and cationic (FRET-K) substrates, respectively. Fluorescence output of reaction mixtures with free OaAEP1-C247A (0.05 μM) or patchwork cage cpAaLS-OaAEP1-C247A/AaLS-13 (1.3 μM) for (B) FRET-N and FRET-K. The reactions were performed at 25 °C and pH 7.0. Reaction mixtures were excited at 350 nm and fluorescence measured at 450 nm. The data reported are normalized to the fluorescence intensity of the substrate peptides in the reaction buffer.

A fluorescence kinetic assay for the caged OaAEP1-C247A was developed based on previously established protocols (Fig. 6A).^28,38^ The substrate peptide included an OaAEP1-C247A recognition sequence, 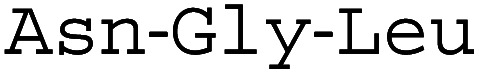, as well as a FRET pair, Abz fluorophore at the N-terminus and a Dnp quencher on the ε-amine of a lysine residue near the C-terminus. Substrate processing by OaAEP1-C247A to form the enzyme–substrate intermediate, results in the separation of the FRET pair, leading to an increase in fluorescence intensity. A large excess (10 equiv.) of the tripeptide, 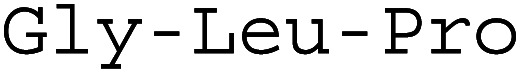, was added to enable the peptide ligation step, thereby completing the catalytic cycle. Replacing the C-terminal amide group in FRET-N with a Lys_6_ tag resulted in FRET-K, which was employed to examine the substrate sorting capacity of the encapsulated PAL system. An increase in fluorescence intensity was observed when FRET-N and FRET-K were incubated with the cpAaLS-OaAEP1-C247A/AaLS-13 complex and the tripeptide 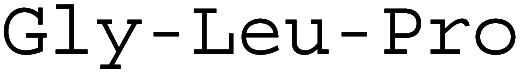, suggesting that the encapsulated PAL is active (Fig. 6B). The peptide ligation activity of the caged PAL was found to be significantly lower than the free enzyme, as shown by the difference in fluorescence output. Based on the band intensity ratio from the SDS-PAGE (Fig. 4B) and loading capacity of AaLS-13 (72 molecules of TEVp-GFP(+36) per capsid),^29^ we estimated that the ratio of cpAaLS-OaAEP1-C247A to AaLS-13 is 1 : 3.3. Accordingly, the turnover frequencies of the caged PAL were approximately 0.050 and 0.038 s^−1^ for FRET-N and -K, respectively. These constants were 28- and 37-fold lower than the *k*_cat_ constants of free OaAEP1-C247A (1.4 s^−1^).^12^ Furthermore, the similar rate constants between FRET-N and FRET-K suggested a lack of substrate sorting (Fig. 6B).

## Conclusion

The engineered lumazine synthase technologies were adapted to create a caged PAL with the aim to create a substrate-sorting organelle that resembles those found in nature. The engineered variant, AaLS-13, assembles with a negatively charged interior which offers a simple strategy for loading charged guest molecules. A substrate sorting system has been previously established using TEVp-GFP(+36) and positively charged peptide substrates.^38^ Unfortunately, significant protein truncation was observed when this tagging strategy was applied to OaAEP1-C247A, which may have resulted from proteolysis during the gene expression or inherent activity of the PAL. An alternative encapsulation strategy was adopted using the circularly permutated AaLS variant (cpAaLS), generating a patchwork cage compartmentalized with PAL. While the caged PAL was shown to be active, its activity was significantly lower than that of the free enzyme. Provided the recombinant fusion OaAEP1-C247A construct was also found in the cell lysate pellet, its poor activity could be caused by its incorrect folding and instability, as the PAL gene was expressed without the cap domain and also fused to cpAaLS. Furthermore, the patchwork cage was found to lack substrate sorting capacity.

For future work, we aim to create a fusion PAL variant resilient to truncation such that it can be encapsulated by AaLS-13 through electrostatic interactions. Indeed, we have recently identified a OaAEP1-C247A variant with an altered residue (D29E) to prevent self-cleavage, thereby improving its purity and activity during purification.^12^ To further enhance folding and stability, the cap domain can be co-produced during gene expression and subsequently removed during cage assembly, possibly by introducing a site-specific protease cleavage tag in the centre of the cap domain. Alternatively, other PALs (*e.g.* butelase 1)^15^ or different transpeptidases, including sortase and omniligase,^39,40^ can be examined. Once a more active encapsulated peptide ligation system is achieved, it is best to undertake structural biology characterization using cryo-electron microscopy,^29^ generating insights that can aid in the understanding and refining of the design of an artificial organelle system for peptide and protein modifications.

## Experimental

All reagents and antibiotics were purchased from Fisher Scientific, unless stated otherwise. Peptide substrates were purchased from ISCA Biochemicals and GenScript. Calcium chloride and isopropyl-β-d-thiogalactopyranoside (IPTG) were purchased from Melford. 30% acrylamide/bis-acrylamide solution and SYBR Safe were purchase from Merck Sigma-Aldrich. Custom oligonucleotides were purchased from Merck Sigma-Aldrich. Buffers, nucleotide triphosphate, and PrimeSTAR HS DNA polymerase were purchased from Takara. All restriction enzymes were purchased from ThermoFisher Scientific. NEBuilder HiFi DNA assembly master mix was purchased from New England Biolabs. Copper sample grids (carbon film 400 mesh) for TEM were purchased from TAAB.

Plasmids containing AaLS-13, cpAaLS and GFP(+36)-TEVp^27,28,32^ were obtained courtesy of a generous donation from Professor Don Hilvert and the Hilvert group at ETH, Zurich.

With the exception of gravity flow columns used for Ni-NTA, protein purifications by column chromatography were performed using an ÄKTA purifier or ÄKTA start systems with stationary phase columns from GE Healthcare. Protein concentrations were estimated by measuring the absorbance at 280 nm (*A*_280_) using Nanodrop UV/Vis spectroscopy (Thermo Scientific), with absorbance baseline correction at 350 nm. The corresponding extinction coefficients at 280 nm (*ε*_280_) and molecular weight (MW) were computed by inputting the amino acid sequence into Expasy-ProtParam (https://web.expasy.org/protparam/). Concentration (*c*) in mol L^−1^ and mg mL^−1^ were calculated using eqn (1) and (2), respectively.

1 *A*_280_ = *ε*_280_ × *c* × *l*

2 *c*(in mg mL^−1^) = *c*(in mol L^−1^) × MW

### Primers

**Table:** 

P1	GCGGCTCCGGCGGCTCGAGCGCGCGTGACGGTGATTATCTGC
P2	AGCCGGATCAGCTGACTAGATTACGGGATGCTCGCGCACG
P3	CGTGCGCGAGCATCCCGTAATCTAGTCAGCTGATCCGGCTGCTAAC
P4	AGATAATCACCGTCACGCGCGCTCGAGCCGCCGGAGC
P5	AACTTTAAGAAGGAGATATACCATGGCGCGTGACGGTGATTATCTGCAC
P6	GGATCCCGGGTCGTTCGCCGGGTTGC
P7	GGCAGCAACCCGGCGAACGACCCGGGATCCATGGCTAGCAAAGGTGAACGTCTGTTTCG
P8	TGGTGGTGGTGGTGCTCGAGCTTGTAGCGTTCGTCGCGTCC
P9	ACAAGGTACCCGGTTAATCTAGTCAGCTGATCCGGCTGCTAAC
P10	ACCGGGTACCTTGTAGCGTTCG
P11	ATAATTGGATCCAGCGTGGGTACCCGTTGG
P12	AATTAACTCGAGTTACGGGATGCTCGCGCACG
P13	TAACTCGAGTAAGCGGCGAACGATG
P14	ACTCGAGTTAGTCGTTCGCCGGGTTGC
P15	ATAATTGGATCCGGAGAAAGCTTGTTTAAGGGGCCGC
P16	CCGTGCTCGAGTTAATTCATGAGTTGAGTCGCTTCCTTAACTGGC

### Molecular cloning

#### His_6_-GFP(+36)-OaAEP1-C247A

The plasmid pACYC-His-GFP(+36)-OaAEP1-C247A was used for the IPTG-inducible production of the N-terminal His_6_ tagged GFP(+36)-OaAEP1-C247A and was constructed by subcloning the gene encoding OaAEP1-C247A^12,14^ (using primers P1 and P2), into a pACYC vector derived from the GFP(+36)-TEVp plasmid^28^ (amplified by PCR, using primers P3 and P4) by Gibson assembly. The sequence of the newly assembled plasmids was confirmed by DNA Sanger sequencing performed by Eurofins Genomics.

#### OaAEP1-C247A-GFP(+36)-His6

The plasmid pET28b-OaAEP1-C247A-GFP(+36)-His was used for the IPTG-inducible production of the C-terminal His_6_ tagged OaAEP1-C247A-GFP(+36). It was constructed by subcloning the gene encoding OaAEP1-C247A (P5 and P6), and gene encoding GFP(+36)^28,32,37^ (P7 and P8) into a pET28b vector by Gibson assembly. The sequence of the newly assembled plasmids was confirmed by DNA Sanger sequencing performed by Eurofins Genomics.

#### His_6_-GFP(+36)

The plasmid pET28b-His-GFP(+36) was used for preparing N-terminal His_6_ tagged GFP(+36) by IPTG induction. It was constructed by PCR amplification using primers P9 and P10. The PCR product was digested by DpnI (ThermoFisher) then transformed into DH5α *E. coli*. Sequences of the product plasmids were confirmed by DNA Sanger sequencing performed by Eurofins Genomics.

#### cpAaLS-OaAEP1-C247A

The plasmid pACTet-cpAaLS-OaAEP1-C247A was used for the tetracycline-inducible production of the cpAaLS-OaAEP1-C247A. The plasmid was constructed in two steps. Initially, the gene encoding for the full length OaAEP1-C247A was subcloned (primers P11 and P12) into a pACTet vector in frame with the gene encoding for cpAaLS by Gibson assembly. Then, the DNA sequence encoding for the cap domain of OaAEP1-C247A was deleted using primers P13 and P14. The sequence of all plasmids was confirmed by DNA Sanger sequencing performed by Eurofins Genomics.

#### cpAaLS-TEVp

The plasmid pACTet-cpAaLS-TEVp was used for the tetracycline-inducible production of the cpAaLS-TEVp. The TEVp gene was amplified from pACYC-His6-GFP(+36)-TEVp using PCR and primers P15 and P16. The PCR was treated with restriction enzymes BamHI and XhoI then inserted into the pACTet-cpAaLS-OaAEP1-C247A plasmid (also treated with BamHI and XhoI) by sticky ends T4 DNA ligation. The sequence of all plasmids was confirmed by DNA Sanger sequencing performed by Eurofins Genomics.

### Amino acid sequence of the construct cloned

#### 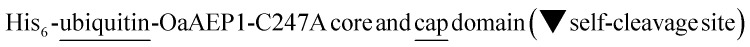


MGMAHHHHHHM̲Q̲I̲F̲V̲K̲T̲L̲T̲G̲K̲T̲I̲T̲L̲E̲V̲E̲P̲S̲D̲T̲I̲E̲N̲V̲K̲A̲K̲I̲Q̲D̲K̲E̲G̲I̲P̲P̲D̲Q̲Q̲R̲L̲I̲F̲A̲G̲K̲Q̲L̲E̲D̲G̲R̲T̲L̲S̲D̲Y̲N̲I̲Q̲K̲E̲S̲T̲L̲H̲L̲V̲L̲R̲L̲R̲G̲G̲ARDGD▼YLHLPSEVSRFFRPQETNDDHGED▼SVGTRWAVLIAGSKGYANYRHQAGVCHAYQILKRGGLKDENIVVFMYDDIAYNESNPRPGVIINSPHGSDVYAGVPKDYTGEEVNAKNFLAAILGNKSAITGGSGKVVDSGPNDHIFIYYTDHGAAGVIGMPSKPYLYADELNDALKKKHASGTYKSLVFYLEACESGSMFEGILPEDLNIYALTSTNTTESSWAYYCPAQENPPPPEYNVCLGDLFSVAWLEDSDVQNSWYETLNQQYHHVDKRISHASHATQYGNLKLGEEGLFVYMGSNPANDN̲Y̲T̲S̲L̲D̲G̲N̲A̲L̲T̲P̲S̲S̲I̲V̲V̲N̲Q̲R̲D̲A̲D̲L̲L̲H̲L̲W̲E̲K̲F̲R̲K̲A̲P̲E̲G̲S̲A̲R̲K̲E̲E̲A̲Q̲T̲Q̲I̲F̲K̲A̲M̲S̲H̲R̲V̲H̲I̲D̲S̲S̲I̲K̲L̲I̲G̲K̲L̲L̲F̲G̲I̲E̲K̲C̲T̲E̲I̲L̲N̲A̲V̲R̲P̲A̲G̲Q̲P̲L̲V̲D̲D̲W̲A̲C̲L̲R̲S̲L̲V̲G̲T̲F̲E̲T̲H̲C̲G̲S̲L̲S̲E̲Y̲G̲M̲R̲H̲T̲R̲T̲I̲A̲N̲I̲C̲N̲A̲G̲I̲S̲E̲E̲Q̲M̲A̲E̲A̲A̲S̲Q̲A̲C̲A̲S̲I̲P̲

#### 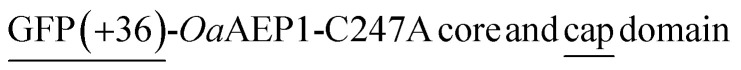


MHHHHHHGSGMA̲S̲K̲G̲E̲R̲L̲F̲R̲G̲K̲V̲P̲I̲L̲V̲E̲L̲K̲G̲D̲V̲N̲G̲H̲K̲F̲S̲V̲R̲G̲K̲G̲K̲G̲D̲A̲T̲R̲G̲K̲L̲T̲L̲K̲F̲I̲C̲T̲T̲G̲K̲L̲P̲V̲P̲W̲P̲T̲L̲V̲T̲T̲L̲T̲Y̲G̲V̲Q̲C̲F̲S̲R̲Y̲P̲K̲H̲M̲K̲R̲H̲D̲F̲F̲K̲S̲A̲M̲P̲K̲G̲Y̲V̲Q̲E̲R̲T̲I̲S̲F̲K̲K̲D̲G̲K̲Y̲K̲T̲R̲A̲E̲V̲K̲F̲E̲G̲R̲T̲L̲V̲N̲R̲I̲K̲L̲K̲G̲R̲D̲F̲K̲E̲K̲G̲N̲I̲L̲G̲H̲K̲L̲R̲Y̲N̲F̲N̲S̲H̲K̲V̲Y̲I̲T̲A̲D̲K̲R̲K̲N̲G̲I̲K̲A̲K̲F̲K̲I̲R̲H̲N̲V̲K̲D̲G̲S̲V̲Q̲L̲A̲D̲H̲Y̲Q̲Q̲N̲T̲P̲I̲G̲R̲G̲P̲V̲L̲L̲P̲R̲N̲H̲Y̲L̲S̲T̲R̲S̲K̲L̲S̲K̲D̲P̲K̲E̲K̲R̲D̲H̲M̲V̲L̲L̲E̲F̲V̲T̲A̲A̲G̲I̲K̲H̲G̲R̲D̲E̲R̲Y̲K̲V̲P̲G̲GSGGSGGSGGSGGSSARDGD▼YLHLPSEVSRFFRPQETNDDHGED▼SVGTRWAVLIAGSKGYANYRHQAGVCHAYQILKRGGLKDENIVVFMYDDIAYNESNPRPGVIINSPHGSDVYAGVPKDYTGEEVNAKNFLAAILGNKSAITGGSGKVVDSGPNDHIFIYYTDHGAAGVIGMPSKPYLYADELNDALKKKHASGTYKSLVFYLEACESGSMFEGILPEDLNIYALTSTNTTESSWAYYCPAQENPPPPEYNVCLGDLFSVAWLEDSDVQNSWYETLNQQYHHVDKRISHASHATQYGNLKLGEEGLFVYMGSNPAND▼N̲Y̲T̲S̲L̲D̲G̲N̲A̲L̲T̲P̲S̲S̲I̲V̲V̲N̲Q̲R̲D̲A̲D̲L̲L̲H̲L̲W̲E̲K̲F̲R̲K̲A̲P̲E̲G̲S̲A̲R̲K̲E̲E̲A̲Q̲T̲Q̲I̲F̲K̲A̲M̲S̲H̲R̲V̲H̲I̲D̲S̲S̲I̲K̲L̲I̲G̲K̲L̲L̲F̲G̲I̲E̲K̲C̲T̲E̲I̲L̲N̲A̲V̲R̲P̲A̲G̲Q̲P̲L̲V̲D̲D̲W̲A̲C̲L̲R̲S̲L̲V̲G̲T̲F̲E̲T̲H̲C̲G̲S̲L̲S̲E̲Y̲G̲M̲R̲H̲T̲R̲T̲I̲A̲N̲I̲C̲N̲A̲G̲I̲S̲E̲E̲Q̲M̲A̲E̲A̲A̲S̲Q̲A̲C̲A̲S̲I̲P̲

#### 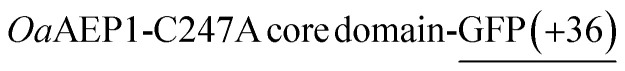


MARDGD▼YLHLPSEVSRFFRPQETNDDHGED▼SVGTRWAVLIAGSKGYANYRHQAGVCHAYQILKRGGLKDENIVVFMYDDIAYNESNPRPGVIINSPHGSDVYAGVPKDYTGEEVNAKNFLAAILGNKSAITGGSGKVVDSGPNDHIFIYYTDHGAAGVIGMPSKPYLYADELNDALKKKHASGTYKSLVFYLEACESGSMFEGILPEDLNIYALTSTNTTESSWAYYCPAQENPPPPEYNVCLGDLFSVAWLEDSDVQNSWYETLNQQYHHVDKRISHASHATQYGNLKLGEEGLFVYMGSNPANDPGSMA̲S̲K̲G̲E̲R̲L̲F̲R̲G̲K̲V̲P̲I̲L̲V̲E̲L̲K̲G̲D̲V̲N̲G̲H̲K̲F̲S̲V̲R̲G̲K̲G̲K̲G̲D̲A̲T̲R̲G̲K̲L̲T̲L̲K̲F̲I̲C̲T̲T̲G̲K̲L̲P̲V̲P̲W̲P̲T̲L̲V̲T̲T̲L̲T̲Y̲G̲V̲Q̲C̲F̲S̲R̲Y̲P̲K̲H̲M̲K̲R̲H̲D̲F̲F̲K̲S̲A̲M̲P̲K̲G̲Y̲V̲Q̲E̲R̲T̲I̲S̲F̲K̲K̲D̲G̲K̲Y̲K̲T̲R̲A̲E̲V̲K̲F̲E̲G̲R̲T̲L̲V̲N̲R̲I̲K̲L̲K̲G̲R̲D̲F̲K̲E̲K̲G̲N̲I̲L̲G̲H̲K̲L̲R̲Y̲N̲F̲N̲S̲H̲K̲V̲Y̲I̲T̲A̲D̲K̲R̲K̲N̲G̲I̲K̲A̲K̲F̲K̲I̲R̲H̲N̲V̲K̲D̲G̲S̲V̲Q̲L̲A̲D̲H̲Y̲Q̲Q̲N̲T̲P̲I̲G̲R̲G̲P̲V̲L̲L̲P̲R̲N̲H̲Y̲L̲S̲T̲R̲S̲K̲L̲S̲K̲D̲P̲K̲E̲K̲R̲D̲H̲M̲V̲L̲L̲E̲F̲V̲T̲A̲A̲G̲I̲KHGRDERYKLEHHHHHH

#### 
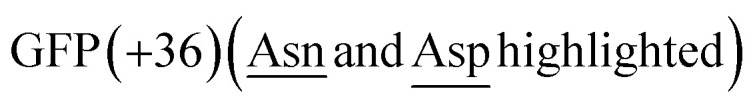


MHHHHHHGSGMASKGERLFRGKVPILVELKG**D̲**V**N̲**GHKFSVRGKGKG**D̲**ATRGKLTLKFICTTGKLPVPWPTLVTTLTYGVQCFSRYPKHMKRH**D̲**FFKSAMPKGYVQERTISFKK**D̲**GKYKTRAEVKFEGRTLV**N̲**RIKLKGR**D̲**FKEKG**N̲**ILGHKLRY**N̲**F**N̲**SHKVYITA**D̲**KRK**N̲**GIKAKFKIRH**N̲**VK**D̲**GSVQLA**D̲**HYQQ**N̲**TPIGRGPVLLPR**N̲**HYLSTRSKLSK**D̲**PKEKR**D̲**HMVLLEFVTAAGIKHGR**D̲**ERYKVPG

#### AaLS-13

MEIYEGKLTAEGLRFGIVASRFNHALVGRLVEGAIDCIVRHGGREEDITLVCVPGSWEIPVAAGELARKEDIDAVIAIGVLIEGAEPHFDYIASEVSKGLANLSLELRKPISFGDITDDELEEAIECAGTEHGNKGWEAALSAIEMANLFKSLRLEHHHHHH

#### 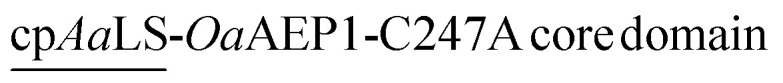


M̲T̲L̲E̲Q̲A̲I̲E̲R̲A̲G̲T̲K̲H̲G̲N̲K̲G̲W̲E̲A̲A̲L̲S̲A̲I̲E̲M̲A̲N̲L̲F̲K̲S̲L̲R̲G̲T̲G̲G̲S̲G̲S̲S̲M̲E̲I̲Y̲E̲G̲K̲L̲T̲A̲E̲G̲L̲R̲F̲G̲I̲V̲A̲S̲R̲F̲N̲H̲A̲L̲V̲D̲R̲L̲V̲E̲G̲A̲I̲D̲C̲I̲V̲R̲H̲G̲G̲R̲E̲E̲D̲I̲T̲L̲V̲R̲V̲P̲G̲S̲W̲E̲I̲P̲V̲A̲A̲G̲E̲L̲A̲R̲K̲E̲D̲I̲D̲A̲V̲I̲A̲I̲G̲V̲L̲I̲R̲G̲A̲T̲P̲H̲F̲D̲Y̲I̲A̲S̲E̲V̲S̲K̲G̲L̲A̲N̲L̲S̲L̲E̲L̲R̲K̲P̲I̲T̲F̲G̲V̲I̲T̲A̲D̲AGGAGGSSVGTRWAVLIAGSKGYANYRHQAGVCHAYQILKRGGLKDENIVVFMYDDIAYNESNPRPGVIINSPHGSDVYAGVPKDYTGEEVNAKNFLAAILGNKSAITGGSGKVVDSGPNDHIFIYYTDHGAAGVIGMPSKPYLYADELNDALKKKHASGTYKSLVFYLEACESGSMFEGILPEDLNIYALTSTNTTESSWAYYCPAQENPPPPEYNVCLGDLFSVAWLEDSDVQNSWYETLNQQYHHVDKRISHASHATQYGNLKLGEEGLFVYMGSNPAND

### Enzyme and protein preparation

#### OaAEP1-C247A

Recombinant expression of OaAEP1 in *E. coli*, BL21(DE3), was performed following the protocol reported previously.^12,14^

#### His_6_-GFP(+36)-OaAEP1-C247A

Recombinant expression was performed in *E. coli*, BL21(DE3). Gene expression was induced by adding IPTG (0.4 mM) at 16 °C for 18 h. The cell pellet from a 1 L culture was suspended in a lysis buffer containing 50 mM sodium phosphate (pH 7.4), 2 M NaCl, lysozyme (0.1 mg mL^−1^), DNase I (5 μg mL^−1^), RNase A (5 μg mL^−1^) and PMSF (35 μg mL^−1^). After lysis by sonication, and clearance by centrifugation (27 000*g*, 4 °C, 15 min), the supernatant was loaded onto 2 mL of Ni-NTA resin (Bio-Rad) in a gravity flow column (Bio-Rad). The column was washed three times with lysis buffer containing 10 mM imidazole (20 mL each), the remaining bound protein was eluted with lysis buffer containing 300 mM imidazole. Ni-NTA elution fractions containing the desired protein were combined and concentrated using an Amicon Ultra-15 centrifugal filter unit (10 kDa MWCO) (Merck Millipore). The concentrated protein was then purified through a SEC column (Superdex 200, GE Healthcare) that had been pre-equilibrated in sodium phosphate (pH 7.4), 2 M NaCl. Protein containing fractions were analysed by SDS-PAGE.

#### OaAEP1-C247A-GFP(+36)-His_6_

Recombinant expression was performed in *E. coli*, BL21(DE3). Gene expression was induced by adding IPTG (0.4 mM) at 16 °C for 18 h. The cell pellet from a 1 L culture was suspended in a lysis buffer containing 50 mM sodium phosphate (pH 7.4), 2 M NaCl, lysozyme (0.1 mg mL^−1^), DNase I (5 μg mL^−1^), RNase A (5 μg mL^−1^) and PMSF (35 μg mL^−1^). After lysis by sonication, and clearance by centrifugation (27 000*g*, 4 °C, 15 min), the supernatant was loaded onto 2 mL of Ni-NTA resin (Bio-Rad) in a gravity flow column (Bio-Rad). The column was washed three times with lysis buffer containing 10 mM imidazole (20 mL each), the remaining bound protein was eluted with lysis buffer containing 300 mM imidazole. Ni-NTA elution fractions were analysed by SDS-PAGE.

#### His_6_-GFP(+36)

Recombinant expression was performed in *E. coli*, BL21(DE3). Gene expression was induced by adding IPTG (0.1 mM) at 16 °C for 20 h. The cell pellet from a 500 mL culture was suspended in a lysis buffer containing 50 mM sodium phosphate (pH 7.4), 2 M NaCl, lysozyme (0.1 mg mL^−1^), DNase I (5 μg mL^−1^). After lysis by sonication, and clearance by centrifugation (27 000*g*, 4 °C, 15 min), the supernatant was loaded onto 2 mL of Ni-NTA resin (Bio-Rad) in a gravity flow column (Bio-Rad). The column was washed three times with lysis buffer containing 10 mM imidazole (15 mL each), the remaining bound protein was eluted with lysis buffer containing 300 mM imidazole. Ni-NTA elution fractions containing the desired protein were concentrated using an Amicon Ultra-15 centrifugal filter unit (10 kDa MWCO) (Merck Millipore). The concentrated protein was then purified through a SEC column (Superdex 75, GE Healthcare) that had been pre-equilibrated in sodium phosphate (pH 7.4), 200 mM NaCl, 5 mM EDTA. Protein containing fractions were analysed by SDS-PAGE. Protein concentration was estimated by UV-Vis measuring the absorbance at 488 nm (*ε*_488_ = 36 600 M^−1^ cm^−1^).

#### cpAaLS-OaAEP1-C247A/AaLS-13 patchwork protein cage

Recombinant expression was performed in *E. coli*, SHuffle T7 Express, which was doubly transformed with a pACYC plasmid containing the codon optimised gene for cpAaLS-OaAEP1-C247A and a pMG plasmid containing the codon optimized gene for AaLS-13.^27^ Gene expression was induced by adding IPTG (0.1 mM) and tetracycline (0.1 μg mL^−1^) at 16 °C for 18 h. The cell pellet from a 1 L culture was suspended in a lysis buffer containing 50 mM sodium phosphate (pH 8.0) 300 mM NaCl, lysozyme (0.1 mg mL^−1^), DNase I (5 μg mL^−1^), RNase A (5 μg mL^−1^) and PMSF (35 μg mL^−1^). After lysis by sonication, and clearance by centrifugation (27000*g*, 4 °C, 15 min), the supernatant was loaded onto 2 mL of Ni-NTA resin (Bio-Rad) in a gravity flow column (Bio-Rad). The column was washed three times with lysis buffer containing 10 mM imidazole (20 mL each), the remaining bound protein was eluted with lysis buffer containing 300 mM imidazole. Ni-NTA elution fractions containing the desired protein were combined, then concentrated and buffer exchanged into 50 mM sodium phosphate (pH 7.0), 200 mM NaCl, 5 mM EDTA using an Amicon Ultra-15 centrifugal filter unit (30 kDa MWCO) (Merck Millipore). The NaCl concentration was then increased to 600 mM by adding 5 M NaCl. The protein was then purified through a SEC column (Superpose 6 GL increase 16/600, GE Healthcare) that had been pre-equilibrated in 50 mM sodium phosphate (pH 7.0) 150 mM NaCl, 1 mM EDTA, 1 mM TCEP-HCl. Fractions containing the desired protein were combined, then analysed by SDS-PAGE and TEM. Total protein concentration was calculated using the extinction coefficient of AaLS-13. Enzyme concentration was then calculated by assuming a 1 : 100 enzyme to cage protein ratio.

#### cpAaLS-TEVp/AaLS-13 patchwork protein cage

Recombinant expression was performed in *E. coli*, BL21(DE3), which was doubly transformed with a pACYC plasmid containing the codon optimized gene for cpAaLS-TEVp and a pMG plasmid containing the codon optimized gene for AaLS-13. Gene expression was induced by adding IPTG (0.1 mM) and tetracycline (0.1 μg mL^−1^) at 16 °C for 18 h. The cell pellet from a 500 mL culture was suspended in a lysis buffer containing 50 mM sodium phosphate (pH 8.0) 300 mM NaCl, lysozyme (0.1 mg mL^−1^), DNase I (5 μg mL^−1^). After lysis by sonication, and clearance by centrifugation (27000*g*, 4 °C, 15 min), the supernatant was loaded onto 2 mL of Ni-NTA resin (Bio-Rad) in a gravity flow column (Bio-Rad). The column was washed three times with lysis buffer containing 10 mM imidazole (15 mL each), the remaining bound protein was eluted with lysis buffer containing 300 mM imidazole. Ni-NTA elution fractions containing the desired protein were combined, then concentrated and buffer exchanged into 50 mM sodium phosphate (pH 7.4) 200 mM NaCl, 5 mM EDTA using an Amicon Ultra-15 centrifugal filter unit (30 kDa MWCO) (Merck Millipore). The NaCl concentration was then increase to 600 mM by adding 5 M NaCl. The protein was then purified through a SEC column (superpose 6 GL increase 16/600, GE Healthcare) that had been pre-equilibrated in 50 mM sodium phosphate (pH 7.0) 200 mM NaCl, 5 mM EDTA. Fractions containing the desired protein were combined, then analysed by SDS-PAGE. Total protein concentration was calculated using the extinction coefficient of AaLS-13. Enzyme concentration was then calculated by assuming a 1 : 100 enzyme to cage protein ratio.

#### Negative stain transmission electron microscopy

Glow discharged copper grids, 400 mesh, covered with carbon (TAAB) were incubated face down on a 10 μL droplet of purified protein samples (0.05–0.15 mg mL^−1^) for 1 min. The grids were then washed three times with 10 μL droplets of H_2_O, followed by two incubations on 10 μL droplets of uranyl acetate for 20 s. All imaging was conducted using a JOEL 2100-JEM transmission electron microscope, operated at 200 kV. Images were processed using the Gatan software.

#### GFP(+36) stability assay

The purified protein GFP(+36) was incubated with or without OaAEP1-C247A in 50 mM MES buffer (pH 6.0) with 50 mM NaCl, 1 mM EDTA and 0.5 mM TCEP. The reaction mixtures had a total volume of 100 μL, with final concentrations of GFP(+36) at 4 μM and OaAEP1-C247A at either 2 or 0 μM. The mixture was incubated at 20 °C for 22 h then analysed by SDS-PAGE.

#### Fluorescent peptide assay for cpAaLS-OaAEP1-C247A activity

Stock solution of the FRET substrate peptides (10 mM) and GLP (100 mM) were prepared by dissolving in dH_2_O. Peptides were added to free or encapsulated enzymes (0.05 μM and 0.3 μM, respectively) in 50 mM sodium phosphate buffer (pH 7.0) containing 200 mM NaCl, 5 mM EDTA in a 96-well plate. The reaction mixtures had a total volume of 100 μL, with final concentrations of FRET substrates, GLP and enzymes at 400 μM, 4 mM and 400 nM, respectively. Time-dependent fluorescence of the reaction mixtures was monitored at 25 °C with a 350–10 nm excitation filter and an 450–10 nm emission filter on a FLUOstar Omega microplate reader (BMG Labtech).

Product concentration was calculated using fluorescence relative to the final fluorescence value where all FRET quenching pairs are separated (eqn (3)).3
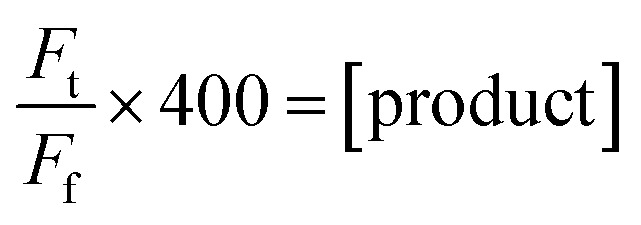


Change in product concentration over the first 150 min was used to estimate the initial rate (*k*) by fitting the data to a linear regression. Turnover frequency was estimated using eqn (4), with total enzyme concentration (*E*) assuming a host : guest ratio of 1 : 3.3.4
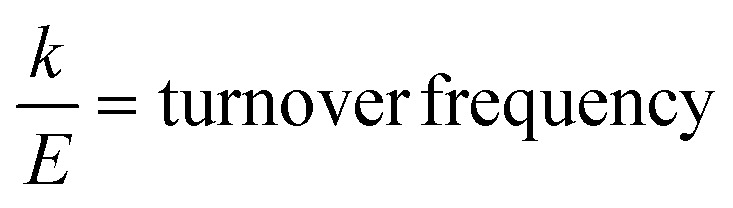


## Conflicts of interest

There is no conflict of interest.
